# Applications of Matrix Metalloproteinase-9-Related Nanomedicines in Tumors and Vascular Diseases

**DOI:** 10.3390/pharmaceutics17040479

**Published:** 2025-04-07

**Authors:** Xuying Li, Zhuping Xu

**Affiliations:** Department of Ophthalmology, West China Hospital, Sichuan University, Chengdu 610041, China; lixy1121@163.com

**Keywords:** matrix metalloproteinase-9, nanomedicine, targeted drug delivery, cancer, vascular diseases, metastasis, angiogenesis

## Abstract

Matrix metalloproteinase-9 (MMP-9) is implicated in tumor progression and vascular diseases, contributing to angiogenesis, metastasis, and extracellular matrix degradation. This review comprehensively examines the relationship between MMP-9 and these pathologies, exploring the underlying molecular mechanisms and signaling pathways involved. Specifically, we discuss the contribution of MMP-9 to tumor epithelial–mesenchymal transition, angiogenesis, and metastasis, as well as its involvement in a spectrum of vascular diseases, including macrovascular, cerebrovascular, and ocular vascular diseases. This review focuses on recent advances in MMP-9-targeted nanomedicine strategies, highlighting the design and application of responsive nanoparticles for enhanced drug delivery. These nanotherapeutic strategies leverage MMP-9 overexpression to achieve targeted drug release, improved tumor penetration, and reduced systemic toxicity. We explore various nanoparticle platforms, such as liposomes and polymer nanoparticles, and discuss their mechanisms of action, including degradation, drug release, and targeting specificity. Finally, we address the challenges posed by the heterogeneity of MMP-9 expression and their implications for personalized therapies. Ultimately, this review underscores the diagnostic and therapeutic potential of MMP-9-targeted nanomedicines against tumors and vascular diseases.

## 1. Introduction

Matrix metalloproteinases (MMPs) constitute a family of calcium-dependent, zinc-containing endopeptidases. These enzymes are initially synthesized as inactive zymogens by connective tissues, such as the endothelium and fibroblasts, as well as by inflammatory cells, including macrophages and neutrophils. These precursors are secreted into the extracellular matrix and subsequently activated through proteolytic cleavage by enzymes such as fibrinolytic enzymes [1]. Currently, the human MMP family comprises 23 identified proteins [2]. Table 1 presents a comprehensive overview of their classification and functional roles, adapted from Wang et al. [3]. Notably, MMP-9, secreted by cells such as neutrophils, macrophages, and endothelial cells, plays a crucial role in the degradation of the extracellular matrix, thereby facilitating tumor invasion and angiogenesis. These unique functions have attracted significant research interest. The *MMP-9* gene, located on chromosome 20q11.1-13.1, encodes a protein composed of four domains: (1) an N-terminal propeptide domain that binds to the catalytic zinc ion and maintains the stability of the pro-MMP-9 zymogen; (2) a catalytic domain containing zinc and calcium ions, which forms the enzymatic active site and includes a hinge region involved in collagen or gelatin binding; (3) a hinge region; and (4) a C-terminal hemopexin-like domain essential for collagenase activity [4,5,6,7]. The MMP-9 promoter binds multiple transcription factors, including NF-κB, AP-1, and SP-1, and its expression is regulated by various signaling pathways. MMP-9 significantly contributes to tumor progression and metastasis through its involvement in tumor angiogenesis, invasion, metastasis, and extracellular matrix degradation [8,9,10]. Furthermore, MMP-9 significantly influences the pathogenesis of vascular diseases such as atherosclerosis and abdominal aortic aneurysm by destabilizing atherosclerotic plaques and degrading the aortic wall [11,12]. As a secreted MMP, MMP-9 is readily accessible to extracellularly targeted nanomedicines, offering advantages over membrane-bound or intracellular MMPs. Its widespread expression in various cancers and vascular diseases has led to its investigation as a biomarker for patient stratification and personalized diagnostics and therapeutics [13,14,15,16].

Tumors are leading causes of global mortality, with approximately 10 million deaths in 2020, according to the World Health Organization (WHO). Lung, colorectal, liver, gastric, and breast cancers account for the majority of cancer-related deaths [17]. Chemotherapy remains a primary treatment for tumors, aiming to control tumor growth, prevent metastasis, and enhance the success rates of solid tumor surgeries while reducing the risk of recurrence. However, conventional chemotherapy drugs for tumors often face limitations such as poor bioavailability, low tissue specificity, high systemic toxicity, and reduced efficacy against highly heterogeneous tumors.

Vascular diseases encompass a diverse range of pathologies affecting the arterial, venous, and lymphatic systems, ultimately compromising tissue function due to impaired perfusion and drainage. The global burden of vascular disease is substantial. Cardiovascular diseases, predominantly including coronary artery disease and stroke, account for an estimated 17.9 million deaths annually [18], with stroke being a major cause of long-term disability [19]. Furthermore, microvascular complications, such as diabetic retinopathy, affect approximately 22% of individuals with diabetes, significantly impacting their quality of life [20]. Current therapeutic strategies for vascular diseases encounter several key challenges. These include the difficulty in achieving adequate drug concentrations at the target lesion, limited drug penetration across the vessel wall, non-specific drug accumulation leading to systemic side effects, and the complex interplay of underlying pathophysiological mechanisms, often necessitating multi-targeted therapeutic interventions.

**Figure 1:**
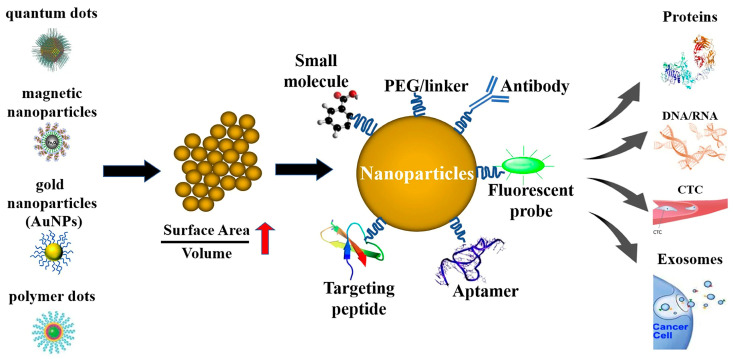
Nanoparticles recognize and bind to cancer-associated molecules, facilitating enhanced tumor diagnosis. Adapted from Zhang et al. [23].

Nanomedicine involves the application of nanotechnology to medicine. It leverages the unique physicochemical properties of materials at the nanoscale to address the current limitations in disease diagnosis and treatment [21]. Nanoparticles, defined as particles with diameters ranging from 1 to 100 nm, possess a high surface area-to-volume ratio, which confers unique physicochemical properties [22]. Nanoparticles have emerged as powerful tools in biomedicine, with applications in cancer therapy, drug delivery, vaccine delivery, gene therapy, the treatment of inflammatory and autoimmune diseases, imaging, diagnostics, tissue engineering, and antibacterial materials. Figure 1, originally published by Zhang et al., illustrates the mechanism by which various nanoparticles recognize and bind to cancer-associated molecules, thereby enhancing tumor diagnosis [23]. Compared with conventional chemotherapeutics, nanodrugs offer the potential for targeted accumulation and controlled release within the tumor microenvironment, enhancing local drug uptake and minimizing systemic side effects. Nanoparticles can be classified based on material and size, as shown in Table 2 and Table 3. The gelatinase activity of MMP-9 has spurred the development of numerous nanocarriers that utilize MMP-9 as a regulatory or responsive target. This review aims to elucidate the regulatory mechanisms of MMP-9 through diverse signaling pathways, consolidate the understanding of MMP-9’s involvement in tumors and vascular diseases, and highlight the recent advancements in MMP-9-targeted nanomedicine strategies, ultimately providing valuable insights into the diagnostic and therapeutic potential of nanomedicines for tumors and vascular diseases.

## 2. Relationship Between MMP-9 and Tumors and Vascular Diseases

### 2.1. Relationship Between MMP-9 and Tumors

#### 2.1.1. Relationship Between MMP-9 and Tumor Epithelial–Mesenchymal Transition

MMP-9, a gelatinase capable of degrading major ECM components including type IV collagen, plays a critical role in the epithelial–mesenchymal transition (EMT) of cancer cells. EMT is the process by which epithelial cells acquire mesenchymal stem cell-like characteristics. This complex process involves the loss of epithelial traits, such as intercellular adhesion and cell polarity, and the gain of mesenchymal properties, including motility and invasiveness [77]. EMT is essential for tumor invasion and progression. It facilitates the detachment of cancer cells from the primary tumor, enabling the infiltration of surrounding tissues (direct infiltration). Furthermore, mesenchymal cancer cells can invade the vasculature, disseminate through the circulatory system, and establish distant metastases through the mesenchymal–epithelial transition (MET), the reverse process of EMT, at secondary sites, where they regain epithelial characteristics [78]. Cells undergoing EMT also acquire stem cell-like properties, contributing to their resistance to chemotherapy and targeted therapies [79]. Additionally, these cells interact with the tumor microenvironment, promoting immunosuppression and metabolic adaptation [80].

MMP-9, through ECM degradation, reduces cell–cell adhesion, promoting cancer cell detachment and invasion. This degradation also facilitates cancer cell entry into the vasculature [9]. Furthermore, ECM degradation triggers the release of factors such as TGF-β and VEGF, which can lead to the downregulation of E-cadherin and the upregulation of N-cadherin, the hallmarks of EMT [81].

#### 2.1.2. Relationship Between MMP-9 and Tumor Angiogenesis and Metastasis

MMP-9 also promotes angiogenesis, facilitating tumor growth and intravasation, ultimately leading to distant metastasis [8]. MMP-9 overexpression is closely linked to tumor angiogenesis and invasive metastatic behavior [10].

MMP-9 plays a significant role in vasculogenic mimicry and tumor invasion, impacting glioma patient survival [82]. Du et al. demonstrated that MMP-9 secreted by glioblastoma cells enhances VEGF utilization by bone marrow-derived CD45^+^ cells, promoting angiogenesis and regulating tumor invasion [83]. Long et al. found that melanoma induces MMP-9 and IL-10 secretion. MMP-9 increases vascular permeability, while IL-10 promotes immunosuppression. These effects contribute to the recruitment of granulocytic myeloid-derived suppressor cells (MDSCs) to the lungs, creating a pre-metastatic niche and facilitating melanoma lung metastasis [84]. Furthermore, non-VEGF-driven intussusceptive angiogenesis, observed in human melanoma metastases with high MMP-9 expression and immune cell infiltration near intravascular pillars, is rare in mouse patient-derived xenografts (PDXs). MMP-9 inhibition suppresses intravascular pillar formation [85]. Bruno et al. observed a persistent elevation of MMP-9, its inhibitors TIMP-1/2, and angiopoietin in colorectal cancer, unlike in intestinal inflammatory diseases. The TIMP-1/2/MMP-9 axis correlates with angiogenesis and invasion, and MMP-9 inhibits natural killer (NK) cell function, thereby regulating tumor metastasis [86]. Therefore, targeting MMP-9 is crucial for research on anti-tumor angiogenesis, invasion, and metastasis.

### 2.2. Relationship Between MMP-9 and Vascular Diseases

Many factors, including infection, hyperlipidemia, hyperglycemia, surgery, and autoimmune diseases [87], have the potential to impair the vascular wall and even induce neovascularization. MMP-9 levels and related signaling pathways are closely related to vascular pathologies. MMP-9’s gelatinase activity degrades key extracellular matrix components such as collagen and elastin, disrupting vascular wall integrity, including the blood–brain and blood–retinal barriers [88]. MMP-9 also plays a role in the formation and progression of atherosclerotic plaques [12] and promotes thrombosis [89], contributing to cardiovascular events. In the process of MMP-9-mediated vascular damage, various factors such as inflammation and hypoxia upregulate the levels of pro-angiogenic factors including VEGF and FGF. Consequently, endothelial cells are activated, leading to neovascularization [90].

#### 2.2.1. Relationship Between MMP-9 and Macrovascular Diseases

The chronic overexpression of MMP-2/9 in vascular smooth muscle cells and macrophages within the infrarenal aorta degrades the elastic matrix, weakening vascular wall elasticity and contributing to abdominal aortic aneurysm development [91,92]. Wang et al. demonstrated that VEGF significantly enhances MMP-9 expression in vascular smooth muscle cells via its receptor flt-1, promoting vascular basement membrane degradation, smooth muscle cell migration, and angiogenesis, ultimately contributing to atherosclerosis [93].

#### 2.2.2. Relationship Between MMP-9 and Cerebrovascular Diseases

MMP-9 plays a significant role in the pathogenesis of various cerebrovascular diseases, with elevated MMP-9 levels consistently correlating with poor clinical prognosis [94,95]. The blood–brain barrier (BBB), comprising the cerebral microvascular endothelium, pericytes, and astrocytes, is vulnerable to disruption under pathological conditions [96]. For instance, Polavarapu et al. demonstrated that the intracerebral injection of tumor necrosis factor-like weak inducer of apoptosis (TWEAK) activates MMP-9 and the upstream NF-κB pathway, thereby increasing BBB permeability [97]. Similarly, interleukin-1β (IL-1β) also induces MMP-9 secretion and the degradation of tight junction proteins in pericytes. Conversely, melatonin has been shown to protect the BBB integrity by regulating Notch-3 expression, the NF-κB pathway, and NF-κB nuclear translocation, resulting in the downregulation of MMP-9 and the preservation of tight junction proteins [98]. Furthermore, Machida et al. reported that thrombin, acting through its receptor PAR-1 and the PKCθ-Akt and PKCδ-ERK1/2 pathways, triggers substantial MMP-9 release from pericytes, leading to BBB dysfunction [99]. Chen et al. demonstrated that the glutamate receptor NMDAR upregulates MMP-9 expression in mouse cerebrovascular endothelium via ERK1/2 phosphorylation, disrupting occludin in the BBB [100]. In ischemic stroke, reactive nitrogen species, such as nitric oxide and peroxynitrite, activate MMPs by downregulating caveolin-1 and activating nitric oxide synthase, thereby exacerbating BBB damage and ischemia/reperfusion injury. This highlights MMPs as potential therapeutic targets [101]. Metformin has been shown to protect the BBB in a mouse model of middle cerebral artery occlusion by downregulating the JNK pathway, reducing MMP-9 expression, and preserving the tight junction protein ZO-1, ultimately reducing infarct size [102]. Subarachnoid hemorrhage also induces MMP-9 expression and activation. Increased cyclophilin A in pericytes activates the NF-κB pathway and MMP-9 via the CD147 receptor, disrupting tight junctions [103]. Conversely, glucocorticoids prevent intracranial hemorrhage in preterm infants by inhibiting MMP-9 and caspase-3 activity in the lateral ventricular germinal matrix of fetal animals [104].

#### 2.2.3. Relationship Between MMP-9 and Ocular Vascular Diseases

MMP-9 is a critical protein in ocular research, particularly concerning fundus diseases, due to its activity against type IV collagen, a major component of retinal endothelial cell basement membranes and Bruch’s membrane [105]. In a rat model of diabetes, elevated MMP-9 levels, decreased vascular endothelial-cadherin, and increased retinal vascular permeability were observed within two weeks [106]. Kowluru et al. demonstrated that high glucose increases MMP-9 expression in cultured retinal endothelial cells by inhibiting sirtuin 1 activity via oxidative stress and enhancing p65 (NF-κB subunit) binding to the *MMP-9* promoter, contributing to diabetic retinopathy [107]. Furthermore, Mishra et al. found that MMP-9 enters the mitochondria in human diabetic retinopathy endothelial cells via HSP70-mediated mechanisms, damaging mitochondrial DNA, disrupting mitochondrial membranes, releasing cytochrome c, and ultimately inducing apoptosis [108]. In retinal endothelial cells, high glucose activates H-Ras, leading to increased NF-κB and MMP-9 expression and accelerated capillary cell apoptosis. Simvastatin, an HMG-CoA reductase inhibitor, inhibits H-Ras membrane anchoring, downregulating its activity and modulating MMP-9 expression [109]. Lambert et al. observed an increased MMP-9 expression five days after laser-induced choroidal neovascularization (CNV) in mice, coinciding with macrophage infiltration and Bruch’s membrane rupture [105]. In a mouse model of laser-induced CNV, *MMP-2/9* double knockout mice exhibited minimal CNV compared to single knockouts, suggesting a synergistic effect of MMP-2 and MMP-9 [110]. Additionally, Kinoshita et al. found that oral genistein administration reduced MMP-9 and ICAM-1 levels in the RPE–choroidal complex in a mouse model of laser-induced CNV, inhibiting neovascularization [111].

Given MMP-9’s crucial role in tumor progression—driving EMT, angiogenesis, growth, and metastasis—and its involvement in the pathogenesis of vascular diseases, the development of MMP-9-related nanomedicines represents a promising therapeutic avenue.

## 3. Signal Pathways Regulating MMP-9 Expression

**Figure 2:**
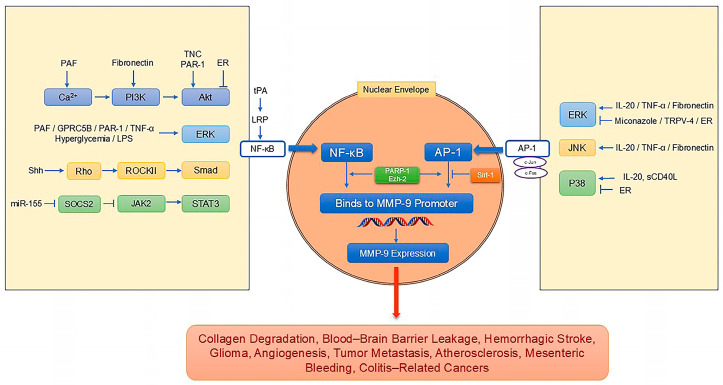
Regulatory network of signaling pathways modulating MMP-9 expression in tumor progression and vascular disease. This illustration describes the complex interplay of signaling pathways that regulate MMP-9 expression and contribute to the development and progression of tumors and vascular diseases.

MMP-9 expression is regulated by a variety of signaling pathways, summarized in Table 4 and Figure 2. Figure 2 illustrates the regulatory network of these pathways on tumor progression and vascular diseases.

The nuclear transcription factor NF-κB directly promotes *MMP-9* gene transcription by binding to its promoter. Consequently, various signaling pathways can regulate MMP-9 expression through NF-κB. For example, platelet-activating factor (PAF) activates NF-κB via the Ca^2+^/PI3K and ERK signaling pathways, upregulating MMP-9 expression [112].

The transcriptional activator AP-1, composed of c-Fos and c-Jun heterodimers, also binds to the *MMP-9* promoter. Hyperglycemia-induced inhibition of the deacetylase sirtuin 1 leads to AP-1 hyperacetylation and increased *MMP-9* promoter binding, promoting MMP-9 expression. Conversely, sirtuin 1 activation downregulates MMP-9 [113].

Poly(ADP-ribose) polymerase-1 (PARP-1) forms complexes with transcription factors such as NF-κB and AP-1 within the *MMP-9* promoter region to enhance transcription [108]. Mishra et al. demonstrated that PARP-1 inhibition in high-glucose-cultured retinal microvascular endothelial cells reduces NF-κB and AP-1 promoter binding, decreasing MMP-9 expression. Notably, PARP-1 is also regulated by sirtuin 1-mediated acetylation [108].

Mitogen-activated protein kinases (MAPKs), including JNK, ERK1/2, and p38 MAPK, play crucial roles in regulating MMP-9 expression [115]. Interleukin-20 (IL-20) treatment of human endothelial cells induces JNK, ERK1/2, and p38 MAPK phosphorylation, promoting the transcription of various factors, including MMP-9, and stimulating angiogenesis [114]. In human umbilical vein endothelial cells (HUVECs), TNF-α upregulates ERK1/2 phosphorylation and downstream AP-1 activation mediated by endoplasmic reticulum protein disulfide isomerase (Endo-PDI) overexpression, increasing MMP-9 transcription and angiogenesis [115]. The antifungal drug miconazole prevents hemorrhagic stroke in zebrafish and mesenteric hemorrhage in mammals by downregulating ERK phosphorylation and MMP-9 expression [117]. Conversely, the knockout of tumor vascular transient receptor potential vanilloid 4 (TRPV4) upregulates ERK phosphorylation and MMP-9 expression, promoting tumor angiogenesis and metastasis [10]. Similarly, under TNF-α, high glucose, or lipopolysaccharide (LPS) stimulation, the overexpression of the orphan C-family G protein-coupled receptor 5B (GPRC5B) phosphorylates ERK1/2, activates NF-κB, and enhances MMP-9 expression in the vascular wall, contributing to inflammation and atherosclerosis [118]. However, Miyoshi et al. found that JNK, but not ERK or p38 MAPK, inhibition almost completely suppressed TNF-α-induced MMP-9 expression in human endothelial cells [116]. The soluble CD40 ligand promotes MMP-9 secretion and angiogenesis in endothelial progenitor cells via the p38 MAPK pathway [119].

The PI3K/Akt pathway also regulates MMP-9 expression and is associated with vasculogenic mimicry in invasive tumors. Tenascin-C (TNC) promotes Akt phosphorylation, upregulating MMP-9 expression and facilitating vasculogenic mimicry and glioma invasion [82]. Jin et al. discovered that fibronectin induces MMP-9 secretion through the JNK, ERK, and PI3K/Akt pathways, mediated by AP-1, leading to collagen degradation [120]. In contrast, Bhowmik et al. demonstrated that estrogen receptor pathway activation in MCF7 breast cancer cells inhibits ERK, p38 MAPK, and PI3K/Akt signaling, decreasing NF-κB and MMP-9 expression, thereby exhibiting an anti-metastatic effect [121].

Thrombin and its receptor protease-activated receptor 1 (PAR-1) activate pericytes, stimulating PKC signaling and promoting MMP-9 secretion via the PKCθ/Akt and PKCδ/ERK pathways, affecting the blood–brain barrier integrity [99]. In mammalian endothelial cells, PKC-α inhibitors reduce ERK1/2 activation and MMP-9 secretion [122].

Rho signaling acts upstream of MMP-9. Renault et al. showed that the morphogen Sonic Hedgehog (Shh) regulates angiogenesis in endothelial cells by upregulating proteins such as MMP-9 through Rho and Rho-associated protein kinase (ROCK) [123]. Chatterjee et al. demonstrated that ROCKII facilitates Smad nuclear localization, upregulates MMP-9, and promotes tumor angiogenesis and lung metastasis in mouse melanoma [124].

The histone methyltransferase Ezh2 regulates MMP-9. Under high glucose conditions, increased Ezh2 activity in retinal endothelial cells leads to *MMP-9* promoter methylation, upregulating MMP-9 transcription and triggering mitochondrial damage and apoptosis [125]. However, during normal mouse embryogenesis, Ezh2 inhibits MMP-9 activation, protecting vascular integrity [126].

Melanoma-derived exosomal miR-155 downregulates suppressor of cytokine signaling 1 (SOCS1), an inhibitor of the JAK2/STAT3 pathway, activating JAK2/STAT3 signaling, promoting MMP-9 expression, and enhancing tumor angiogenesis [127]. In a mouse stroke model, tissue plasminogen activator (tPA) binds to low-density lipoprotein receptor-related protein (LRP), upregulating MMP-9 expression in brain microvascular endothelial cells and causing cerebral hemorrhage [128].

Table 5 summarizes the pathways relevant to the relationship between MMP-9 and tumor progression and vascular diseases.

## 4. Nanomedicine and MMP-9-Targeted Nanoparticles

Nanomedicine leverages nanotechnology—the manipulation of materials at the nanoscale (1 to 100 nm)—to advance medical diagnosis and treatment. Nanoparticles, characterized by their high surface area-to-volume ratio, possess unique physicochemical properties that make them powerful tools in biomedicine.

### 4.1. Comparison of MMP-9-Targeted and Non-Targeted Nanomedicines

Non-targeted nanomedicines can enhance drug solubility, prolong circulation time, and improve chemotherapeutic efficacy while mitigating systemic toxicity through the enhanced permeability and retention (EPR) effect. However, tumor heterogeneity often limits their efficacy, hindering optimal accumulation within deep tumor tissues. In contrast, MMP-9-targeted nanomedicines employ active targeting, binding to overexpressed MMP-9 in the tumor microenvironment. This approach enhances specificity and local penetration, improves therapeutic outcomes, and potentially overcomes chemotherapy resistance [137]. Nonetheless, heterogeneous MMP-9 expression necessitates personalized diagnostic and treatment strategies based on individual MMP-9 biomarker profiles [14,15,16]. Furthermore, the design and synthesis of MMP-9-targeted nanoparticles are more complex than their non-targeted counterparts.

### 4.2. MMP-9 Overexpression and MMP-9-Responsive Nanoparticles

MMP-9, frequently overexpressed in certain cancer types, plays a crucial role in tumor microenvironment remodeling. Many nanomedicines exploit MMP-9 overexpression to modify their physical properties, achieving targeted controlled release. For example, MMP-9 cleavage can facilitate deeper tumor penetration and enhance permeability [138]. Smaller nanoparticles, generated through this cleavage, can be internalized via endocytosis rather than phagocytosis [139]. The nanoparticle surface charge is also critical; a positive charge facilitates cellular penetration and uptake through interaction with the negatively charged cell membrane [140]. Furthermore, the nanomedicine composition significantly impacts drug delivery. Hydrophobic drugs are often encapsulated within a hydrophobic core. Upon exposure to MMP-9, this core can be exposed, triggering drug release [141].

### 4.3. Mechanisms of Action of MMP-9-Responsive Nanoparticles

In general, MMP-9-responsive nanoparticles operate through several mechanisms: degradation, drug release, and targeting specificity.

#### 4.3.1. Degradation

(a) Direct cleavage: Nanoparticles composed of MMP-9-cleavable materials, such as gelatin, are degraded in MMP-9-rich environments such as tumor microenvironments and vasculopathies, facilitating local drug release [139,141].
(b) Cross-linking disruption: MMP-9-sensitive linkers maintain nanoparticle stability. Overexpressed MMP-9 disrupts these linkages, destabilizing the structure and releasing the encapsulated drug [137,141].
(c) Matrix degradation: Nanoparticles embedded within the ECM are released upon MMP-9-mediated matrix degradation [142].

#### 4.3.2. Drug Release

(a) Degradation-triggered release: MMP-9 activity degrades the nanoparticles, altering their composition, size, and cross-linking density, thereby modulating drug release rates [139].
(b) Enhanced permeability: MMP-9 increases tumor blood vessel and tissue permeability, enhancing nanoparticle penetration into the tumor tissues [142,143].

#### 4.3.3. Targeting Specificity

(a) Active targeting: Nanoparticles decorated with MMP-9-targeting ligands (e.g., antibodies, peptides, small molecules) bind to specific regions such as the active site of MMP-9 [144,145,146], enhancing the accumulation at MMP-9 overexpression sites and minimizing the off-target effects.
(b) Passive targeting: Nanoparticles passively accumulate in tumors due to the EPR effect, exploiting leaky tumor vasculature and impaired lymphatic drainage [147].

### 4.4. Heterogeneity of MMP-9 Expression and Targeted Therapy Efficacy

MMP-9 expression varies considerably across tumors and vascular diseases, impacting targeted nanomedicine efficacy. Tumors with a high MMP-9 expression (e.g., breast, glioblastoma, colorectal, melanoma, and lung cancers) are particularly amenable to targeted therapies [143,144,146,148]. In tumors with a moderate MMP-9 expression (e.g., prostate, pancreatic, and head and neck cancers), nanoprobes and other diagnostic tools can assess MMP-9 levels to guide personalized strategies [149], and MMP-9-targeted nanomedicines may still impede the disease progression. However, in lesions with a low MMP-9 expression, the potential for significant off-target effects limits the utility of MMP-9-targeted therapies.

## 5. MMP-9-Related Nanomedicines in Tumor Angiogenesis and Metastasis

### 5.1. MMP-9-Targeted Nanomedicine in Anti-Tumor Angiogenesis and Metastasis Therapy

Liposomes represent one of the most clinically advanced nanodrug delivery systems. Gao et al. modified cationic liposomes with low-molecular-weight gelatin (an MMP-9 substrate) to reduce the interstitial fluid pressure, thereby improving drug delivery and enabling the MMP-9-dependent release of doxorubicin, quercetin, and imatinib. This approach significantly promoted apoptosis and inhibited metastasis in breast cancer cells [143]. Similarly, Han et al. designed cationic liposomes modified with an MMP-9-cleavable peptide (OMPE), a glutamate-rich fragment, and hydrophobic oleic acid, forming anionic nanohybrids. MMP-9 cleavage reversed the surface charge to cationic, enhancing drug endocytosis [140]. Zhang et al. constructed hybrid nanovesicles with surfaces composed of cancer cell membranes and MMP-9-responsive liposome membranes. Within the tumor microenvironment, MMP-9 activation of the nanovesicle surface peptides increased their uptake, and they subsequently released siRNAs and chemotherapeutic drugs to effectively inhibit non-small cell lung cancer growth [150]. Deng et al. designed liposomal spherical nucleic acids (SNAs) composed of DOPE, adriamycin, CpG, and MMP-9-responsive peptides. In the MMP-9- and glutathione-rich tumor microenvironment, the MMP-9-mediated degradation of the peptides released chemotherapeutic drugs, enhancing dendritic cell activation, T cell expansion, and inhibiting tumor growth [151].

Polymer nanoparticles offer flexible composition and structure, enabling the encapsulation of hydrophobic drugs for solubilization and drug loading via chemical conjugation. This multifunctional delivery approach enhances controlled release and reduces chemotherapeutic toxicity. Porta et al. constructed paclitaxel-loaded PDMS–PMOXA polymerosomes with surfaces modified with MMP-9-cleavable SRL peptides. These polymerosomes accumulated in the liver and tail vein of zebrafish embryos, significantly reducing tumor cells in MCF7-transplanted embryos [147]. Battistella et al. prepared an amphiphilic diblock copolymer comprising a hydrophilic MMP-9-responsive peptide and a hydrophobic Toll-like receptor agonist. In circulation, the polymer self-assembled into spheres. In the tumor environment, the MMP-9-mediated hydrolysis of the hydrophilic peptide exposed and retained the hydrophobic fragment in the 4T1 tumor. This accumulation formed micron-sized scaffolds, releasing the immunostimulatory drug, increasing pro-inflammatory cytokines (IP-10, MCP-1, IL-6), and inhibiting tumor growth and metastasis [152]. Li et al. developed hyaluronic acid/poly(lactic-co-glycolic acid)-poly(ethyleneimine) nanoparticles (PP-HA/NPs) co-delivering doxorubicin/quercetin, which downregulated Akt phosphorylation and MMP-9 expression, significantly inhibiting breast cancer cell invasion [153]. Jiang et al. designed a sequential delivery strategy for synergistic breast cancer treatment: combretastatin A4 nanoparticles (a vascular disrupting agent) disrupted neovascularization and exacerbated tumor hypoxia, upregulating MMP-9 and subsequently triggering MMP-9-activated doxorubicin-loaded nanoparticles to enhance chemotherapy targeting [145]. Poly(N-vinylpyrrolidone)-block-poly(ε-caprolactone) (PVP-b-PCL) nanoparticles loaded with tetrandrine downregulated MMP-2/9 expression and upregulated TIMP-3 via a “Trojan strategy,” effectively inhibiting non-small cell lung cancer cell invasion [154]. Low-molecular-weight heparin-tocopheryl succinate (TOS) nanoparticles inhibited G-MDSC-produced MMP-9 and prevented early lung recruitment of tumor-induced G-MDSCs, impeding tumor invasion and colonization [84]. Yu et al. designed MMP-2/9-sensitive, folate receptor-targeting nanoparticles with a sandwich structure. The upregulation of MMP-2/9 in the tumor environment removed the PEG shell, exposing folate, enhancing nanoparticle endocytosis by B16 melanoma cells, improving drug targeting, and promoting intracellular chemotherapy uptake [146]. α-TOS-loaded self-assembled polymer nanoparticles induced intracellular ROS accumulation and endothelial cell apoptosis, reducing MMP-9 expression and inhibiting angiogenesis and tumor invasion in head and neck squamous carcinoma [155]. Ehrsam et al. constructed self-assembled nanoparticles composed of paclitaxel, hemisuccinic acid, and MMP-9-sensitive peptides, demonstrating MMP-9-dependent cytotoxicity in glioblastoma cells [144]. S-triazine-based dendrimers targeted and inhibited MMP-2/9 activity and could be conjugated with cancer cell-specific ligands and anticancer drugs to inhibit hepatocellular carcinoma growth [156]. Nanovesicles containing MMP-9-sensitive lipopeptide and PEG groups released gemcitabine upon MMP-9- and glutathione-mediated hydrolysis, inhibiting pancreatic ductal carcinoma growth [141]. PLGA–PEG polymer particles with outer long-chain PEG- and MMP-9-cleavable linkers created smaller PLGA–b-PEG nanoparticles for enhanced uptake by pancreatic cancer cells [139]. Doxorubicin-conjugated RGD peptide nanoparticles, responsive to MMP-9 and pH, transformed from spheres to rods in the tumor environment, enhancing penetration and doxorubicin release and inducing apoptosis [138]. MMP-2/9-sensitive TGK enzyme-sensitive polymer nanomicelles exhibited specific degradation, improved stability, and higher drug uptake than mPEG2K–α-TOS nanomicelles, achieving ideal local accumulation and targeted release [157].

Inorganic nanoparticles, offering good dispersibility, stability, and unique electromagnetic and optical properties, are promising for thermal therapy and imaging. Magnetic nanoparticles with a surface-modified glucose oxidase layer amplified oxidative stress, promoting MMP-9 expression and facilitating tumor extracellular matrix degradation, which enhanced nanoparticle penetration and inhibited breast tumor growth [142]. Magnetic heating with methotrexate-coupled magnetic nanoparticles reduced angiogenic signals (MMP-9, VEGF-R1) in bladder tumors, decreasing tumor size, promoting tumor destruction, and preventing recurrence [158]. Gold nanoparticles conjugated with cytotoxic protein NKCT1 inhibited MMP-9 expression and metastasis by suppressing p38 MAPK, ERK1/2, PI3K/Akt phosphorylation and NF-κB nuclear translocation in MCF-7 cells [121]. CuS@mSiO2-PEG photothermal nanoparticles reduced HeLa cell metastasis and enhanced survival in tumor-bearing mice by downregulating MMP-2/9 and inhibiting the non-receptor tyrosine kinase/focal adhesion kinase pathway [159]. Gallic acid-loaded gold nanoparticles inhibited EGF-induced Akt and c-Jun phosphorylation in triple-negative breast cancer, suppressing MMP-9 transcription and hindering invasion and metastasis [160]. Macrophage membrane-camouflaged hollow bismuth selenide nanoparticles loaded with quercetin downregulated Akt phosphorylation and MMP-9 expression, inhibiting breast cancer metastasis [161]. MMP-9-sensitive SNAs modified with avidin on mesoporous silica nanoparticles selectively released cisplatin and bortezomib in Kras-mutant lung tumors, synergistically inducing cell death [148]. Gold nanoparticles inhibited MMP-9 activity in prostate cancer cells, promoting anti-cancer cytokine secretion and exerting cytotoxic effects [162]. Zwitterionic tetrapeptides on nanoparticle surfaces, activated by MMP-9 cleavage, triggered nanoparticle auto-aggregation, enhancing drug uptake [137]. Metallofullerenol Gd@C_82_(OH)_22_ nanoparticles selectively inhibited MMP-9 activity, treating angiogenesis and invasion in pancreatic cancer [163,164]. Han et al. constructed a dual-enzyme-sensitive nanocarrier with a PEG modification for enhanced circulatory stability. In the pancreatic cancer environment, MMP-9 hydrolyzed the PEG, exposing the RGD-targeting ligand. Lysosomal cathepsin B then cleaved the GFLG peptide conjugated with gemcitabine and CdSe/ZnS quantum dots, facilitating drug release and increasing active gemcitabine intracellularly [165]. Carbon quantum dots/Cu_2_O complexes (CQDs/Cu_2_O) specifically reduced MMP-2/9, VEGF, and cytoskeletal component levels, inducing cell death and exhibiting anti-angiogenic effects in SKOV3 ovarian cancer cells [166].

### 5.2. MMP-9-Targeted Assays for Tumor Cells

Numerous studies utilize MMP-9-targeted vectors as nanoprobes for tumor cell detection, diagnosis, and assessment of invasiveness. Liu et al. developed Au@4-mercaptobenzonitrile@Ag@peptide nanoprobes that anchored to tumor cell membranes, weakening the surface-enhanced Raman scattering (SERS) signal on the cell membrane. This enabled SERS sensors to detect MMP-9 secretion during cellular communication and to assess breast and liver cancer cell invasiveness [13]. Furthermore, fluorescent nanoprobes have been constructed by conjugating pH-sensitive fluorescent dyes to nanoparticles, such as Fe_3_O_4_, via MMP-9-responsive peptide chains. MMP-9 cleavage induces fluorescence, enabling the visualization of abnormal pH and MMP-9 overexpression in tumor environments, facilitating tumor invasiveness assessment [14,15,16,167]. Nossier et al. used gelatin to modify colorimetric gold nanoparticles, stabilizing them against aggregation-inducing agents. However, exposure to the bladder cancer marker MMP-2/9 destabilized the nanoparticles, causing a color change. A subsequent interaction with 6-mercaptohexanol (6-MCH) further shifted the color from red to blue, enabling a sensitive, rapid, and non-invasive bladder cancer diagnosis [149]. Black et al. labeled gold nanoparticles with radionuclides I^125^ and In^111^ and modified them with MMP-9 substrate peptides to create a contrast agent for single-photon emission computed tomography (SPECT) imaging [168]. The pharmacokinetics of these nanocontrast agents allows for the observation of MMP-9 expression variations among tumors and the assessment of biological behaviors, including tumor invasiveness [169].

### 5.3. Effect of Tumor Heterogeneity on MMP-9 Targeting Efficacy

Tumor heterogeneity, characterized by diverse cell populations with varying phenotypes and functional traits, contributes to heterogeneous MMP-9 expression [170]. This heterogeneity challenges the effective delivery and distribution of MMP-9-targeted drugs, leading to inconsistent drug efficacy and promoting drug resistance [171]. Moreover, MMP-9’s pivotal role in the tumor microenvironment, including angiogenesis and metastasis, complicates the complete inhibition of MMP-9-associated tumor progression by targeted therapies [10].

## 6. MMP-9-Related Nanomedicines in Vascular Diseases

### 6.1. MMP-9-Related Nanomedicines in Macrovascular Diseases

#### 6.1.1. Nanomedicines Regulating MMP-9 Expression in Macrovascular Diseases

Intimal hyperplasia, characterized by smooth muscle cell aggregation in the vascular wall, can lead to post-angioplasty restenosis [172,173]. Given MMP-9’s role in promoting vascular smooth muscle cell proliferation and migration, various nanomedicines aim to maintain endothelial stability by downregulating MMP-9 expression and related pathways. For instance, a docetaxel-loaded LDL-mimetic lipid nanoparticle significantly reduced the expression of MMP-9, NF-κB, and other proteins in a rabbit model of aortic atherosclerosis, achieving an 80% reduction in the atherosclerotic area compared to the controls [174]. Another study utilized a polyethylene glycol/polyethyleneimine nanoparticle (PEG-Et 1:1/shSmad3) loaded with Smad3 shRNA. This efficiently delivered shRNA to vascular smooth muscle cells, downregulating Smad3 and MMP-9 expression while upregulating the MMP-9 inhibitor TIMP-1, ultimately reducing intimal hyperplasia 14 days post-vascular injury [175]. Chen et al. demonstrated that Foxp1 overexpression in vascular endothelial cells inhibited MMP-9 and cyclin-dependent kinase inhibitor 1B (CDKN1B) expression, which regulates VSM proliferation and neointima formation. They restored intimal homeostasis in a Foxp1 knockout mouse model of femoral artery injury-induced neointima by delivering CDKN1B siRNA-loaded RGD peptide-conjugated magnetic nanoparticles to endothelial cells [176].

#### 6.1.2. MMP-9-Responsive Nanocarriers in Macrovascular Diseases

A gadolinium-rich paramagnetic fluorescent nanomicelle conjugated with the NAP9 peptide, which specifically binds the MMP-9 inducer EMMPRIN, was developed. Combined with magnetic resonance (MR) imaging, this nanomicelle enabled the visualization of EMMPRIN content in the carotid artery wall, allowing the non-invasive analysis of MMP-9 secretion in macrophages and vascular smooth muscle cells post-endarterectomy [177]. Nguyen et al. designed an MMP-9-responsive nanocarrier comprising a brush peptide polymer amphiphile with a specific peptide chain. Intravenously injected, this nanocarrier sensed MMP-9, transforming from discrete spherical particles (15–20 nm) to a grid-like structure, remaining at the infarction site for up to four weeks, and facilitating long-term local accumulation, particularly during vascular leakage and MMP-9 overexpression post-acute myocardial infarction [178].

### 6.2. MMP-9-Related Nanomedicines in Cerebrovascular and Ocular Vascular Diseases

#### 6.2.1. MMP-9-Related Nanomedicines in Cerebrovascular Diseases

Several studies have investigated MMP-9-related nanomedicines in cerebrovascular diseases. Foroshani et al., using a rat model of middle cerebral artery occlusion, found that fullerenol nanoparticles significantly reduced MMP-9 and IL-6 transcription, protected the BBB integrity, and attenuated cerebral edema following ischemia-reperfusion injury [179]. Cai et al. demonstrated that intravenously injected *Momordica charantia*-derived exosome-like nanoparticles (ELNs) delivered miR-5266, downregulating MMP-9, improving the BBB function, reducing infarction area, and mitigating neurological damage in a rat middle cerebral artery occlusion model [180].

#### 6.2.2. MMP-9-Related Nanomedicines in Ocular Vascular Diseases

Nanoparticles targeting MMP-9 in ophthalmology are relatively scarce, with topical administration (eye drops) and intravitreal injections being the primary delivery routes. Miyagawa et al. designed GEH-RGD NP eye drops comprising epigallocatechin-3-gallate (EGCG), RGD peptide, gelatin, and a hyaluronic acid coating. GEH-RGD NPs suppressed HUVEC tube formation and MMP-9 activity in vitro. Daily topical application in a chemical burn-induced corneal neovascularization mouse model reduced MMP-9 and VEGF expression, inhibiting neovascularization [181]. Zeng et al. developed IL-12-PNP, a PLGA nanoparticle encapsulating IL-12, and administered it intravitreally in a diabetic retinopathy mouse model. IL-12-PNP showed superior MMP-9 and VEGF inhibition compared to IL-12 alone in rat endothelial cells in vitro and in vivo, reducing retinal neovascularization and improving retinal thickness [182]. Recently, C_18_PGM, a graphene oxide quantum dot (GOQD) nanoparticle modified with MMP-9-responsive peptide chains and loaded with minocycline, was developed. C_18_PGM was selectively cleaved by MMP-9, releasing minocycline and inhibiting MMP-9 expression in a laser-induced choroidal neovascularization (CNV) mouse model, suppressing choroidal inflammation and neovascularization [183].

#### 6.2.3. Challenges of Blood–Brain Barrier and Blood–Retinal Barrier to Nanodrug Delivery

The BBB and blood–retinal barrier (BRB) stringently regulate molecular passage into brain and retinal tissues, posing significant drug delivery challenges. Tight junctions between endothelial cells restrict paracellular transport and macromolecule diffusion, hindering nanoparticle penetration. Efflux transporters actively restrict drug influx [184]. Additionally, limited and selective receptor-mediated transcytosis further impedes efficient cytoplasmic drug delivery [185].

Strategies to overcome these challenges include focused ultrasound for temporary, localized BBB/BRB disruption [186], intranasal administration for BBB bypass [187], and conjugation with penetrating peptides to enhance cellular uptake and endocytosis [188]. However, careful consideration is needed for potential off-target effects and the optimization of drug dispersion within the target tissue.

## 7. Effects of the Immune System on MMP-9-Targeted Nanoparticles

Systemically administered nanomedicines, typically via intravenous injection, must contend with immune system interactions. Preventing complement system activation is crucial to avoid nanoparticle clearance by the mononuclear phagocyte system [189]. Furthermore, allergic individuals may experience IgE antibody-mediated hypersensitivity reactions [190]. Certain nanoparticles can also induce excessive pro-inflammatory cytokine release, potentially leading to cytokine release syndrome and life-threatening consequences [191].

Combining MMP-9-targeted nanoparticles with immunotherapy holds considerable promise. These combined approaches can stimulate the immune system by modulating the tumor microenvironment [151], delivering relevant molecules [152], and enhancing anti-tumor immune cell infiltration deep within the tumor [192], facilitating tumor cell elimination or immunogenic cell death. MMP-9-targeted therapies can synergistically enhance anti-tumor efficacy as well [192]. Moreover, in vascular diseases where immune-mediated inflammation is central, MMP-9-targeted therapies can deliver anti-inflammatory agents to localized lesions, minimizing systemic side effects [178].

## 8. Conclusions

MMP-9 plays a crucial role in tumor and vascular disease development and progression, offering significant diagnostic and therapeutic potential. Nanomedicine provides innovative strategies to modulate MMP-9 activity and exploit its distribution and enzymatic properties for targeted drug delivery. Despite substantial progress in MMP-9-targeted nanomedicines, further research is needed to optimize their efficacy, safety, and clinical translatability across various organs.

Several challenges remain for nanoparticle-based drugs. First, some nanomaterials, particularly metal nanoparticles, exhibit inherent toxicity [193,194]. Furthermore, poorly biocompatible materials can trigger immune responses and inflammation, including complement activation and hypersensitivity reactions, compromising nanomedicine efficacy [195]. Meticulous material selection is therefore essential, prioritizing biocompatible materials and incorporating biomimetic substances or polyethylene glycol to reduce immunogenicity. Second, nanoparticles face challenges with non-specific uptake, even with targeting, due to factors such as tumor heterogeneity, leading to off-target effects. Refining drug delivery strategies, such as developing stimulus-responsive nanomedicines (as discussed herein), is crucial for enhancing controlled release specificity. Finally, the complex design and synthesis of nanoparticles result in high manufacturing costs. Ensuring drug quality and consistency during large-scale production remains challenging, necessitating comprehensive performance characterization. Clinical trials have demonstrated the feasibility of MMP-9-targeted nanomedicines [196].

Further refinements to MMP-9-targeted nanomedicines can enhance their specificity. Regarding the MMP-9 molecule, the computational design of inhibitors tailored to the specific MMP-9 crystal structure can minimize cross-reactivity with other MMP family members by targeting sites beyond the active region [197]. Developing MMP-9-activated prodrugs can also enhance selectivity and reduce off-target effects [198]. For drug delivery systems, besides stimulus-responsive nanomedicines, synergies with physical modalities (e.g., ultrasound for enhanced drug permeability [186]) and imaging technologies (e.g., MRI and PET for visualizing drug distribution [199]) can be explored. Utilizing exosomes or cell membrane-coated biomimetic nanoparticles can improve lesion permeability and targeting precision while reducing immune-mediated clearance [200]. These combined approaches hold promise for enhancing the selectivity of MMP-9-targeted nanomedicine release, minimizing off-target toxicity, and improving drug utilization.

MMP-9-targeted nanoparticles, as a highly effective drug delivery system, show significant promise when combined with conventional chemotherapy or immunotherapy. They efficiently deliver anti-tumor drugs deep into tumor tissue, enhancing their efficacy and reducing systemic toxicity. Nanotherapy can also mitigate drug resistance mechanisms such as drug efflux and enable the sequential release of multiple chemotherapeutic agents, further reducing the resistance potential.

Ultimately, advancing our understanding of MMP-9 biology and refining nanomedicine delivery systems will be critical for the clinical translation of these promising therapies.

## Figures and Tables

**Table 1:** The structural and functional classification of MMP family proteins (adapted from Wang et al. [3]).

Classification By Function	Types of Human MMPs	Main Substrate	Main Cell Source
Collagenases	1	Collagen, Gelatin	Platelets, Macrophages, Endothelium, Smooth Muscle Cell (SMCs), Fibroblasts
	8	Collagen, Gelatin, Aggrecan	Macrophages, Neutrophils
	13	Collagen, Gelatin, Fibronectin	SMCs, Macrophages, Varicose Veins, Breast Cancer
Gelatinases	2	Platelets, Leukocytes, Endothelium, Vascular Smooth Muscle (VSM), Collagen, Gelatin	Adventitia, Aortic Aneurysm, Varicose Veins
	9	Collagen, Gelatin, Elastin	Microvessels, Macrophages, Neutrophils, Endothelium, VSM, Adventitia, Aortic Aneurysm
Stromelysins	3	Extracellular Matrix (ECM), pro-MMP	Endothelium, Intima, VSM, Platelets, Coronary Artery Disease, Synovial Fibroblasts
	10	ECM, pro-MMP	Atherosclerosis, Uterus, Arthritis, Carcinoma Cells
	11	Insulin-Like Growth Factor Binding Protein, etc.	Brain, Uterus, Angiogenesis
Matrilysins	26	ECM, pro-MMP	Endothelium, Intima, VSM, Uterus
Membrane-Anchored	14	Fibronectin, Laminin, Gelatin	Breast Cancer, Endometrial Tumors
	15	Collagen, pro-MMP-2, pro-MMP-13, Fibronectin	VSM, Fibroblasts, Platelets, Brain, Uterus, Angiogenesis
	16	pro-MMP-2, Fibronectin	Fibroblasts, Leukocytes
	17	pro-MMP-2, Fibronectin	Leukocytes, Angiogenesis
	24	pro-MMP-2	Brain, Breast Cancer
	25	pro-MMP-2, Fibronectin	Leukocytes, Lung, Pancreas, Kidney, Brain, Astrocytoma, Glioblastoma
Metalloelastase	12	Fibronectin, Tenascin-C	Leukocytes, Anaplastic Astrocytomas, Glioblastomas
Enamelysin	20	Elastin, Fibronectin, Laminin	SMCs, Fibroblasts, Macrophages
Epilysin	28	Amelogenin, Dentin Sialophosphoprotein	Tooth Enamel

**Table 2:** Classification of nanoparticles based on material.

Classification by Material	Applications in Medicine	References
Metal Nanoparticles		
Gold Nanoparticles	Drug delivery	[24]
	Cancer treatment	[25]
	Diagnostics	[26]
Silver Nanoparticles	Antimicrobial coatings	[27]
	Wound dressings	[28]
Platinum Nanoparticles	Cancer therapy	[29]
Metal Oxide Nanoparticles		
Titanium Dioxide	Photocatalysis	[30]
Zinc Oxide	Antibacterial agents	[31]
Iron Oxide	Magnetic resonance imaging contrast agents	[32]
	Drug delivery	[33]
Ceramic Nanoparticles		
Silica Nanoparticles	Drug delivery	[34]
	Catalysis	[35]
	Biosensors	[36]
Alumina Nanoparticles	Coating	[37]
Carbon-Based Nanoparticles		
Carbon Nanotubes	Electronics	[38]
	Conductive materials	[39]
	Drug delivery	[40]
Fullerenes	Antioxidants	[41]
	Drug delivery	[42]
Graphene	Flexible electronics	[43]
	Sensors	[44]
Polymeric Nanoparticles		
Poly(Lactic-Co-Glycolic Acid) (PLGA) Nanoparticles	Drug delivery	[45]
	Vaccine delivery	[46]
	Cancer therapy	[47]
Polycaprolactone (PCL) Nanoparticles	Drug delivery	[48]
	Tissue engineering	[49]
	Gene delivery	[50]
Polystyrene (PS) Nanoparticles	Diagnostics	[51]
	Research tools	[52]
Chitosan Nanoparticles	Drug delivery	[53]
	Gene delivery	[54]
	Antimicrobial agents	[55]
Poly(N-Isopropylacrylamide) (PNIPAM) Nanoparticles	Drug delivery	[56]
	Smart materials	[57]
Poly(Methyl Methacrylate) (PMMA) Nanoparticles	Drug delivery	[58]
	Bone cement and other base material	[59]
Dendrimers	Drug delivery	[60]
	Gene delivery	[61]
	Imaging	[62]
Polyethylene Glycol (PEG) Nanoparticles	Drug delivery	[63]
	Protein delivery	[64]
Poly(Alkyl Cyanoacrylate) (PACA) Nanoparticles	Drug delivery	[65]
Hydrogel Nanoparticles	Drug delivery	[66]
	Tissue engineering	[67]
Polypeptide-Based Nanoparticles	Drug delivery	[68]
	Theranostics	[69]

**Table 3:** Classification of nanoparticles based on size.

Classification by Size	Applications in Medicine	References
Quantum Dots (2–10 nm)	Bioimaging	[70]
	Quantum computing	[71]
	Photovoltaics	[72]
Ultra-Fine Particles (1–100 nm)	Catalysis	[73]
	Drug delivery	[74]
	Imaging	[75]
Fine Particles (100–1000 nm)	Coatings	[76]

**Table 4:** The regulatory effects of signaling pathways on MMP-9 expression.

Molecules	Signaling Pathways and Mechanisms	Regulatory Effects on MMP-9 Expression	References
NF-κB	Binding directly to promoter	Upregulation	
Platelet-Activating Factor (PAF)	Ca^2+^/PI3K, ERK pathways	Upregulation	[112]
AP-1	Binding directly to promoter	Upregulation	
Poly ADP Ribosyltransferase-1 (PARP-1)	Formation of transcription complex in promoter	Upregulation	[108]
Sirt-1	Reducing the binding of AP-1 and PARP-1 to promoter	Downregulation	[108,113]
IL-20	JNK, ERK1/2, P38 MAPK pathways	Upregulation	[114]
TNF-α	JNK, ERK1/2, AP-1 pathways	Upregulation	[115,116]
Miconazole	ERK pathway	Downregulation	[117]
Transient Receptor Potential Vanilloid 4 (TRPV-4)	ERK pathway	Downregulation	[10]
G-Protein Coupled Receptor 5B (GPRC5B)	ERK1/2, NF-κB pathways	Upregulation	[118]
Soluble CD40 Ligands	P38 MAPK pathway	Upregulation	[119]
Tenascin-C (TNC)	Akt	Upregulation	[82]
Fibronectin	JNK, ERK, PI3K/Akt pathways, AP-1	Upregulation	[120]
Estrogen Receptor	ERK, P38 MAPK, PI3K/Akt pathways, NF-κB	Downregulation	[121]
Thrombin Receptor (PAR-1)	PKCθ/Akt, PKCδ/ERK pathways	Upregulation	[99]
PKC-A	ERK1/2 pathways	Upregulation	[122]
Sonic Hedgehog (Shh)	Rho, ROCK	Upregulation	[123]
Smads	ROCKII	Upregulation	[124]
Ezh2	Promoting promoter methylation in retinal endothelial cells	Upregulation	[125]
	Inhibiting activation in mouse embryos	Downregulation	[126]
miR-155	SOCS1/JAK2/STAT3 pathway	Upregulation	[127]
Tissue Plasminogen Activator (tPA)	Low-density lipoprotein receptor-associated protein (LRP)	Upregulation	[128]

**Table 5:** Relationship between MMP-9-related signaling pathways and tumor progression or vascular diseases.

MMP-9-Related Signaling Pathways	Corresponding Biological Effects	References
Tumor progression		
VEGF/VEGFR	Stimulation of angiogenesis and tumor growth	[83]
TGF-β	Initially functions as the tumor suppressor, but later promotes metastasis	[129]
EGFR	Enhances tumor cell proliferation and survival	[130]
MAPK	Essential for cell proliferation, differentiation, and survival	[121]
NF-κB	Promotes tumor progression, angiogenesis, and metastasis	[131]
PI3K/Akt/mTOR	Critical for cell growth, survival, and metabolism; frequently dysregulated in cancer	[82]
Wnt/β-catenin	Influences cell proliferation, differentiation, and stem cell renewal; dysregulation can drive tumor development	[132]
Vascular diseases		
TGF-β	Stimulates ECM production, normally restraining vascular smooth muscle cell proliferation; MMP-9 disrupts balance, leading to vascular remodeling and lesion formation	[133]
PDGF	Promotes VSMC migration and proliferation, resulting in neointima formation	[134]
TNF-α	Induces inflammation and ECM restructuring within the vessel wall	[118]
Interleukin	Contributes to the inflammatory response within vascular lesions	[135]
Oxidative stress	Triggers ECM degradation, contributing to vascular damage	[136]

## Data Availability

No new data were created or analyzed in this study. Data sharing is not applicable to this article.

## Abbreviations

The following abbreviations are used in this manuscript:

4T1	mouse mammary tumor cell line
6-MCH	6-hydroxy-1-hexanethiol
Akt	protein kinase B
AP-1	activator protein 1
bFGF	basic fibroblast growth factor
CDKN1B	cycling-dependent kinase inhibitor 1B
CNV	choroidal neovascularization
CpG	5′-C-phosphate-G-3′
CQDs	carbon quantum dots
DOPE, 1	1,2-dioleoyl-sn-glycero-3-phosphoethanolamine
EGCG	epigallocatechin-3-gallate
ELNs	exosome-like nanoparticles
EMMPRIN	extracellular matrix metalloproteinase inducer
EMT	epithelial–mesenchymal transition
ERK	extracellular regulated protein kinases
GFLG	Gly-Phe-Leu-Gly
G-MDSC	granulocyte-like myeloid derived suppressor cells
GOQD	graphene oxide quantum dots
GPRC5B	G protein-coupled receptor 5B
GST	glutathione-S-transferase
HIV	human immunodeficiency virus
HMG-CoA	3-hydroxy-3-methylglutaryl coenzyme A
HSP70	heat shock 70 kda protein
ICAM-1	intercellular adhesion molecule-1
IL-10	interleukin 10
IP-10	interferon–γ–inducible protein 10
JAK2/STAT3	janus kinase 2/signal transduction and activator of transcription 3
JNK	C-jun N-terminal kinase
LDL	low-density lipoprotein
LRP	low density lipoprotein receptor-related protein
MAPK	mitogen-activated protein kinase
MCF7	a breast cancer cell line
MMP	matrix metalloproteinase
mPEG	methoxypolyethylene glycol
NF-κB	nuclear factor kappa-light-chain-enhancer of activated B cells
NKCT1	purified naja kaouthia protein toxin
NMDAR	N-methyl-D-aspartate receptor
PAF	platelet-activating factor
PAR-1	protease-activated receptor 1
PARP-1	poly [adp-ribose] polymerase 1
PDI	protein disulfide isomerase
PDMS–PMOXA	poly(dimethylsiloxane)-poly-b-(methyloxazoline)
PEG	polyethylene glycol
PI3K	phosphoinositide 3-kinase
PKC	protein kinase C
PLGA	poly(lactic-co-glycolic acid)
PP-HA/NPs	hyaluronic acid/poly(lactic acid)-glycolic acid-poly(ethyleneimine) nanoparticles
PTK	poly (5,5-dimethyl-4,6-dithio-propylene glycol azelate)
PVP-b-PCL	poly(N-vinylpyrrolidone)-block-poly(ε-caprolactone)
RGD	L-Arginyl-Glycyl-L-Aspartic acid
ROCK	rho-associated protein kinase
ROS	reactive oxygen species
RPE	retinal pigment epithelium
SERS	surface-enhanced Raman scattering
Shh	sonic hedgehog protein
SKOV3 SMCs	an ovarian cancer cell line smooth muscle cells
SNAs	spherical nucleic acid
SPECT	single-photon emission computed tomography
TAK1	TGF-β-activated kinase 1
TGF-β	transforming growth factor β
TGK	protein-glutamine gamma-glutamyltransferase K
TNF-α	tumor necrosis factor α
TOS	tocopheryl succinate
tPA	tissue plasminogen activator
TRPV4	transient receptor potential cation channel subfamily V member 4
VEGF	vascular endothelial growth factor
VEGF-R1 VSM	vascular endothelial growth factor receptor 1 vascular smooth muscle
ZO	zonula occludens
α-TPGS	α-tocopherol polyethylene glycol succinate
